# Small Bowel Perforation Secondary to Biliary Stent Migration in an Incarcerated Inguinal Hernia

**DOI:** 10.7759/cureus.7268

**Published:** 2020-03-14

**Authors:** Chi Lap Nicholas Tsang, Robert S O'Neill, Christo M Joseph, Tony Palasovski

**Affiliations:** 1 Surgery, The Wollongong Hospital, Wollongong, AUS

**Keywords:** biliary stent, small bowel, general surgery, hernia, perforation

## Abstract

We describe the case of a 90-year-old female who presented with signs of a strangulated inguinal hernia. Further history revealed a paired biliary-pancreatic stent insertion three years prior for ascending cholangitis and a long-standing asymptomatic right inguinal hernia. Biochemistry revealed a slightly elevated C-reactive protein level of 65 mmol/L, but was otherwise unremarkable. Abdominal CT demonstrated two plastic biliary stents within an incarcerated right inguinal hernia. At the time of surgery, a 3-mm perforation due to the stents was identified in the small bowel within the hernia. The stents were retrieved via an enterotomy that was subsequently repaired with full-thickness interrupted sutures. A tissue-suture repair of the inguinal hernia was performed due to significant contamination of enteric contents in the operative field. The patient had an unremarkable recovery and was discharged four days after her operation. This is a very rare acute presentation of stent migration with only a handful of such reported cases in the literature. With the rising number of endoscopic biliary stenting procedures, these complications are likely to increase, and clinicians need to be aware of this possibility in patients with pre-existing hernias.

## Introduction

Biliary stenting is an increasingly common procedure employed in the management of a variety of benign and malignant causes of biliary obstruction. Proximal and distal stent migration are known complications of biliary stenting. It occurs in up to 6% of cases and commonly involves migration into the duodenum, with plastic stents migrating more often than metallic stents [1,2]. Approximately 50% of patients may be asymptomatic of stent migration. Sequelae of stent migration can include symptoms of cholangitis, painless jaundice, bowel perforation (1%), and mortality with reported rates of less than 1% [3-5]. Previous studies have demonstrated that patients with diverticular disease, hernia, or intra-abdominal adhesions are at increased risk of migrated biliary stent-related problems, as intestinal wall thickness and resistance can predispose to localised complications during bowel movement [6,7]. The approach to the management of uncomplicated stent migration can either be endoscopic or surgical, with the latter being the apparent choice for complications including bowel perforation [8,9]. Surgical intervention can range from repair of perforation, bowel resection, and in rare emergent cases, may involve temporary diversion. We present a case of biliary stent migration within an incarcerated inguinal hernia associated with small bowel perforation.

## Case presentation

**Figure 1:**
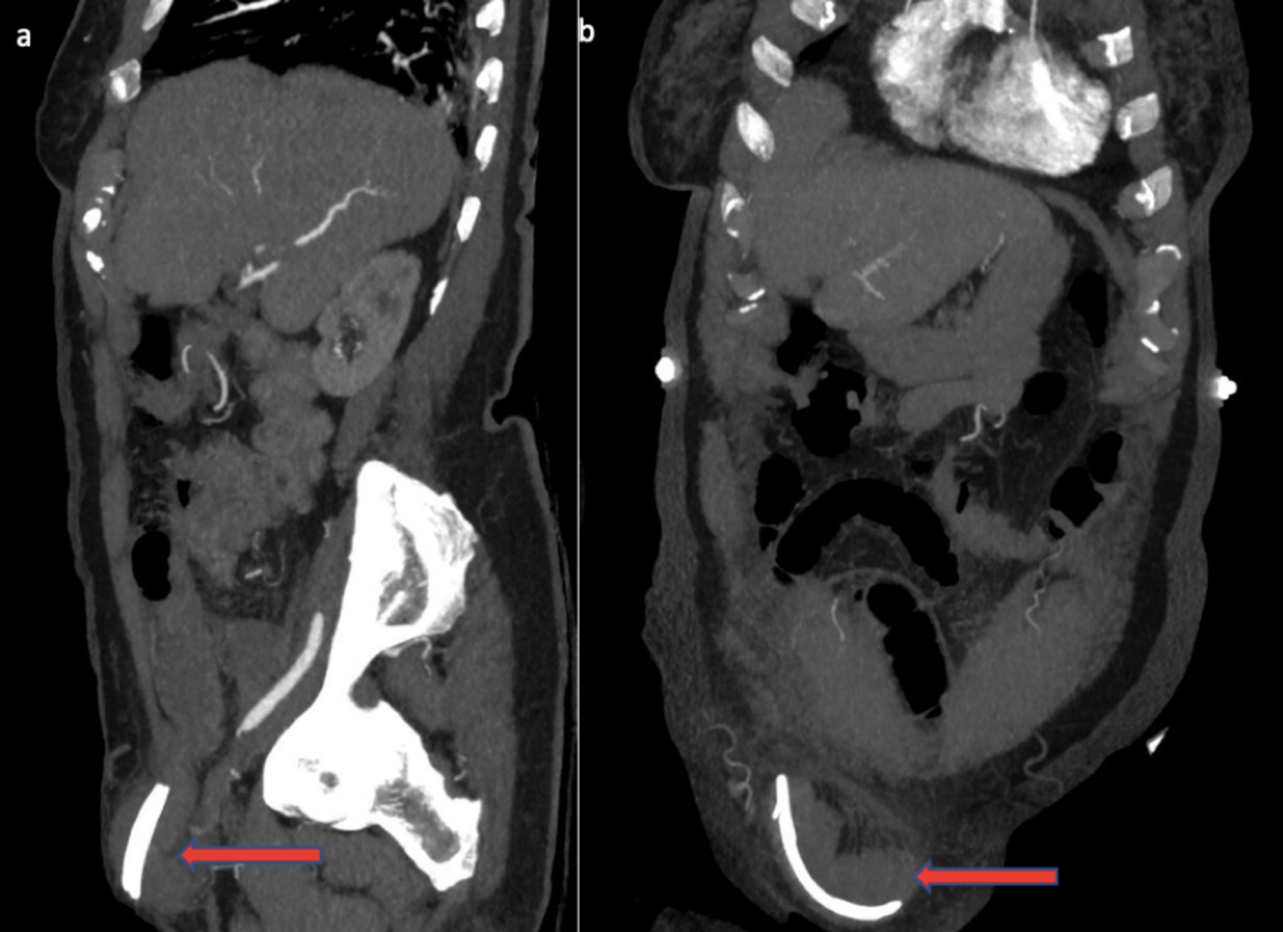
Abdominal CT sagittal (a) and coronal (b) views demonstrating a pair of biliary stents (red arrows) within a bowel containing right inguinal hernia.

**Figure 2:**
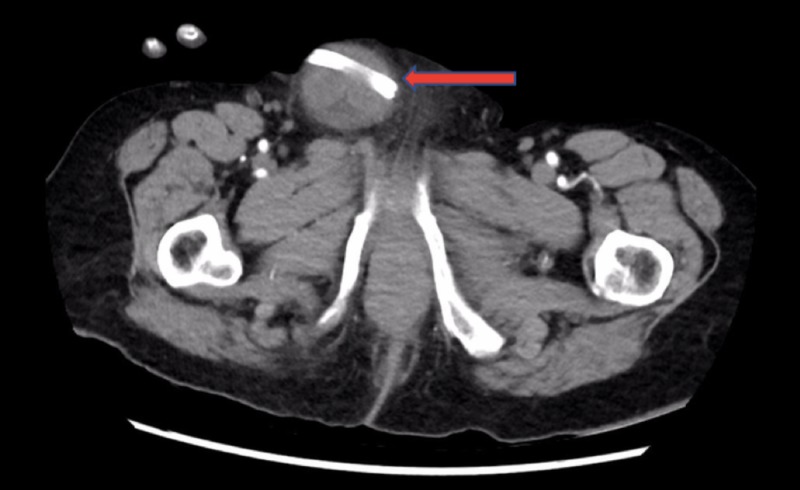
Abdominal CT axial view demonstrating a pair of biliary stents (red arrow) within a bowel containing right inguinal hernia.

A 90-year-old female presented to the emergency department from a high-level care nursing home, complaining of a one-day history of abdominal pain. As the patient was dysphasic due to a previous cerebrovascular accident and had significant impairment in cognition, a reliable history was difficult to ascertain. Her medical background included dementia, atrial fibrillation, a known right inguinal hernia, previous gallstone ileus, ascending cholangitis managed with endoscopic retrograde cholangiopancreatography, biliary sphincterotomy, stone retrieval, and insertion of two 9-cm, 10-Fr straight plastic stents into the bile duct with the ends proximal to cystic duct take-off. Her surgical history included a midline laparotomy and enterotomy for gallstone ileus. Abdominal examination revealed a soft and tender right inguinal mass with overlying erythema consistent with an inguinal hernia concerning for threatened bowel. A pair of tubular structures was palpable within the hernia externally. Biochemical analysis revealed a white cell count of 8.46×10^9^/L and C-reactive protein of 65 mmol/L with a lactate of 1.2 mmol/L. An abdominal CT demonstrated a right-sided inguinal hernia with radiopaque stents within the bowel lumen on sagittal (Figure 1a), coronal (Figure 1b), and axial views (Figure 2).

**Figure 3:**
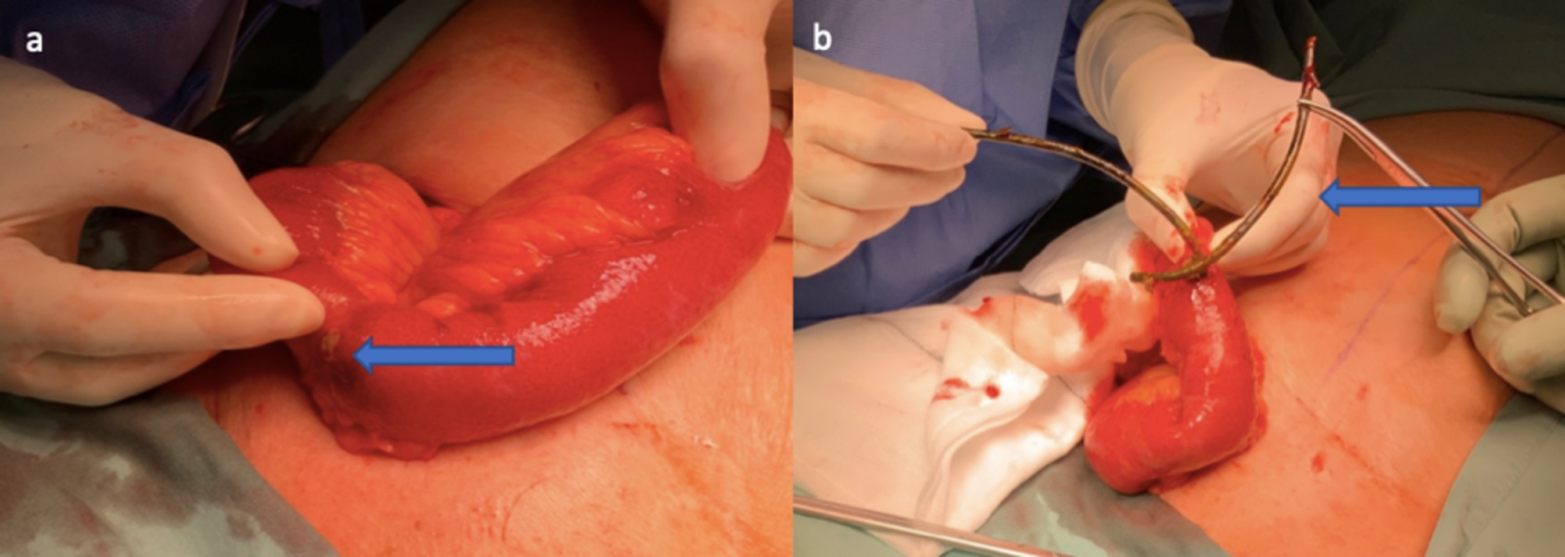
Intra-operative photography demonstrating ileal perforation (a) and retrieval of paired biliary stents from the ileal perforation (b).

An open right inguinal hernia approach was undertaken revealing a thick-walled hernia sac containing an oedematous but viable loop of small bowel within the hernia. Two tubular structures were palpable within the lumen, and a small 3-mm perforation was noted in the small bowel within the hernia with moderate spillage of enteric contents (Figure 3a). The two plastic biliary stents were retrieved via the perforation (Figure 3b). A single layer interrupted full-thickness repair using 3-0 polydioxanone suture (PDS) was used to close the perforation. Repair of the inguinal hernia was conducted by suturing the conjoint tendon to the inguinal ligament using 2-0 PDS. The patient had an unremarkable recovery and was discharged four days after her operation.

## Discussion

Biliary stents can be used to alleviate biliary obstruction secondary to a variety of benign or malignant causes. Complications include stent occlusion, misplacement, migration, and fracture. Biliary stent migration is a well-documented adverse event. Incidence rates of 4.9% and 5.9% were observed for proximal and distal biliary stent migration, respectively. Likewise, incidence rates of 5.2% and 7.5% were observed for proximal and distal pancreatic stent migration, respectively. In addition to this, migration rates for stents placed for benign causes are increased compared to those placed for malignant disease [10,11]. It has been previously demonstrated that migration is more common with plastic stents compared with metallic stents [10,12]. When distal migration does occurs, the stent often passes into stool without issue and is rarely associated with bowel perforation or fistula formation [13]. Risk factors for distal stent migration include benign strictures and ampullary stenosis [10]. The most likely site for perforation is in the duodenum or bowel containing herniae [3,14].

The incidence of stent-related bowel perforation in a pre-existing inguinal hernia is not well documented and is limited to several cases in the literature involving incisional hernias or parastomal hernias [15]. In this case, it was successfully managed surgically, and the patient had an unremarkable postoperative course. 

The management of impacted biliary stents and certainly those involving the possibility of perforation necessitates endoscopic or surgical exploration depending on the location. In this instance, a dual pathology of stent migration into an inguinal hernia was appropriately dealt via an approach for an incarcerated inguinal hernia with biliary stent retrieval via an enterotomy. Mesh was not placed due to an element of enteric contamination and a high likelihood of mesh infection with a reported incidence up to 61.9% [16].

In the presented case, sphincterotomy was performed and a pair of straight plastic stents was placed several years prior due to obstructive choledocholithiasis with associated ascending cholangitis. The stents were not subsequently retrieved due to the advanced age and comorbidities of the patient, and it was thought that even in the event of migration, it would likely pass spontaneously. The risk of complications from stent retrieval did not justify the rare risk of complications associated with stent migration. Although sphincterotomy was performed, its routine implementation during biliary stenting was not recommended as sphincter of Oddi tonus and valves may assist in preventing distal migration of placed stents [10].

There is no strong evidence to advocate for elective hernia repair in those with biliary stenting. Certainly, there are consensus recommendations that advocate for elective removal of biliary stents once they have outlived their usefulness or when migration occurs with signs or symptoms of obstruction or perforation [17]. Some have advocated for abdominal wall herniae to be a relative contraindication to stent placement, although in our clinical scenario, it was appropriately placed as a lifesaving measure [18].

## Conclusions

With placement of biliary stents being more frequently performed, clinicians must be aware of complications of stent migration. As such, clinicians should consider an elective removal or endoscopic retrieval of exchanged biliary stents in patients with a pre-existing herniae, diverticulosis, or adhesions rather than assuming that stents would pass without incident.
